# Predictors of the Outcomes Following the Tocilizumab Treatment for Severe COVID-19

**DOI:** 10.7759/cureus.28428

**Published:** 2022-08-26

**Authors:** Karan Singla, Goverdhan D Puri, Subhrashis Guha Niyogi, Varun Mahajan, Kamal Kajal, Ashish Bhalla

**Affiliations:** 1 Department of Anaesthesia and Intensive Care, Postgraduate Institute of Medical Education and Research, Chandigarh, IND; 2 Department of Internal Medicine, Postgraduate Institute of Medical Education and Research, Chandigarh, IND

**Keywords:** critical care, logistic regression, mortality, tocilizumab, covid-19

## Abstract

Background: Tocilizumab is used in severe COVID-19 yet has significant rates of treatment failure.

Objectives: This retrospective study aimed to identify early predictors of the response to tocilizumab therapy.

Methods: Biochemical and clinical characteristics of adult patients who received tocilizumab for severe COVID-19 pneumonia were retrospectively examined. A multivariable logistic regression model was constructed to identify factors that could predict the failure of tocilizumab therapy. A predictive nomogram was also created using the selected model.

Results: Out of 101 eligible patients, 30 had treatment failure, and 71 survived on a 28-day follow-up. The partial pressure of oxygen to fraction of inspired oxygen ratio (PFR) on the day of tocilizumab administration (100 vs 80.5), lactate dehydrogenase (LDH; 668 vs 507 U/L), neutrophil-to-lymphocyte ratio (NL ratio; 24.7 vs 10), and creatine kinase myocardial band (CKMB; 30.9 vs 22.7 U/L) were significantly different among the non-survivors and survivors, respectively. A logistic regression model was created, identifying LDH, NL ratio, pro-brain natriuretic peptide (ProBNP), and PFR on the day of tocilizumab administration as best predictors of mortality with an optimism-corrected area under the receiver operator characteristics (ROC) curve of 0.82. The model-implied odds ratios for mortality were 1.89 (95% CI 1.13-3.15) for every 100 U/L rise in serum LDH, 2.29 (95% CI 2.2-4.39) for every 10 unit rise in NL ratio, 1.23 (95% CI 0.95-1.58) for every 100 pg/ml increase in ProBNP, and 0.36 (95% CI 0.13-0.95) for every mmHg rise in PFR at intervention.

Conclusion: This study identified NL ratio, LDH, CKMB, and PFR at intervention as important markers of risk of treatment failure following the tocilizumab therapy. A multivariable logistic regression model including LDH, NL ratio, ProBNP, and PFR at intervention best predicted the risk of mortality in patients with severe COVID-19 pneumonia treated with tocilizumab.

## Introduction

As of 25th April 2022, COVID-19 has caused 6,220,390 deaths and 507,501,771 confirmed cases, as reported to the World Health Organization [1]. Though the disease was first reported as far back as December 2019, apart from corticosteroids, the pharmacotherapy for hospitalized patients with COVID-19 pneumonia is still a matter of speculation and much debate. The pathogenesis of severe COVID pneumonia has been attributed to an exaggerated immune response, a “cytokine storm,” which makes the disease amenable to the action of immunomodulatory drugs [2-4]. However, only glucocorticoids have demonstrated a consistent mortality benefit in hospitalized patients on oxygen support (with or without invasive mechanical ventilation) in large randomized controlled trials - the Recovery Trial found mortality of 22.9% in the steroid cohort vs. 25.7% in the usual care cohort [4].

Tocilizumab is a recombinant humanized IgG1 monoclonal antibody, which is directed against both the soluble and the membrane-bound IL-6 receptor. A number of randomized clinical trials have examined the efficacy of tocilizumab in patients with COVID-19 with mixed results. The COVACTA trial found no evidence of clinical improvement [5]. Studies by Stone et al. and Salvarani et al. concluded that tocilizumab was not effective for preventing intubation or death in moderately ill hospitalized patients with COVID-19 [6,7]. However, the EMPACTA (Evaluating Minority Patients with Actemra) and REMAP-CAP (randomized, embedded, multifactorial, adaptive platform trial for community-acquired pneumonia) studies found a significant beneficial effect of tocilizumab [8,9]. A recent meta-analysis demonstrated that tocilizumab reduced mortality by 12%, indicating a number needed to treat of 11. Mortality after tocilizumab treatment was still 27% [10].

As more patients are being treated with this drug, it is important to identify factors that help in the early prediction of the response to tocilizumab therapy, particularly the risk of mortality, i.e., treatment failure following its administration. In this retrospective study, we aimed to study and model the early predictors of treatment failure after tocilizumab therapy.

## Materials and methods

This was a retrospective cohort study done in a single tertiary care center in India. The study protocol was approved by the Institutional Ethics Committee with the approval number of IEC-INT/2022/study-130.

We included all adult patients (>18 years of age), who were admitted to our intensive care unit with severe COVID-19 pneumonia and received one or more doses of tocilizumab in addition to the standard treatment of dexamethasone and low molecular weight heparin. All patients receiving tocilizumab between 1st July 2020 and 31st July 2021 were considered, excluding only the patients with incomplete medical records or those who were transferred to another hospital precluding follow-up.

Severe COVID pneumonia was defined by the presence of at least one of the following: a respiratory rate (RR) ≥ 30 breaths per minute (bpm), peripheral blood oxygen saturation (SpO_2_) ≤ 93%, a PaO_2_/FiO_2_ ratio < 300 mmHg in room air, and lung infiltrates covering more than 50% of lung fields on chest x-rays within 24-48 hours [11].

The decision to administer tocilizumab was taken by a multidisciplinary medical team, taking into account the severity and rapidity of progression of the disease based on clinical and biochemical parameters like oxygen requirement, tachypnea, radiological finding, C-reactive protein (CRP) levels, etc., along with the absence of indicators of active infection such as elevated leukocyte counts, fever, positive cultures or elevated serum procalcitonin, and radiological, microbiological, or molecular evidence of active or latent tuberculosis. Tocilizumab was given at a loading dose of 8 mg/kg intravenously and repeated if there was no effect after 24 hours of the first dose.

Electronic medical records of all eligible adult patients receiving tocilizumab were accessed and fully anonymized for analysis. Demographic, biochemical, and outcome measures were recorded. Patients were divided into survivor cohort and mortality cohort.

Statistical analysis

Data were analyzed in R software version 4.1.2 (R Foundation for Statistical Computing, Vienna, Austria), using tableone, pROC, glmulti, survival, and rms packages. The study parameters were described using mean and standard deviation or median and interquartile range for normally distributed and skewed variables, respectively, after checking for normality by the Shapiro-Wilks test. Categorical variables were described as proportions (%). Holm's correction for multiple comparisons was done, and the adjusted p-values were also reported. A p-value < 0.05 was considered significant. 

Depending on normality, a T-test or Wilcoxon signed-rank test was used for continuous variables as appropriate. Chi-squared test was used for categorical variables. Receiver operating characteristics (ROC) analysis was done to find optimum cut-offs, corresponding specificity and sensitivity, and area under the ROC curve (AUROC) for individual predictors to predict mortality.

For better prediction of mortality, a multivariate logistic regression model was constructed. The optimum model parameters were selected using clinical knowledge, and further by a genetic algorithm-based approach, optimizing the Akaike information criterion. The final model was selected by manual examination from the best candidate models, using backward elimination for sensitivity analysis and bootstrapping in the original population for verification of model performance. A predictive nomogram was also created using the selected model.

## Results

A total of 101 patients with severe COVID-19 treated with tocilizumab were found eligible, and no adult patients receiving tocilizumab needed exclusion due to incomplete electronic medical records or loss to follow-up. Of them, 30 patients died on a 28-day follow-up, and 71 patients survived. The baseline characteristics of the two cohorts are provided in Table 1. The two groups differed in their admission acute physiology and chronic health evaluation (APACHE) and sequential organ failure assessment (SOFA) scores, respiratory rates, partial pressure of oxygen to fraction of inspired oxygen ratio (PFR) on days of admission, drug administration, and intensive care stay. The day of illness when tocilizumab was administered was also different. Serum lactate, D-dimer, pro-brain natriuretic peptide (ProBNP), ferritin, lactate dehydrogenase (LDH), neutrophil-to-lymphocyte ratio (NL ratio), and creatine kinase myocardial band (CKMB) levels differed at admission. However, after Holm's correction for multiple comparisons, PFR on the day of tocilizumab admission, LDH, NL ratio, and CKMB remained significantly different among the two cohorts.

**Table 1:** Baseline and clinical characteristics in our cohort of survivors and non-survivors SD: Standard deviation; IQR: Interquartile range; APACHE II: Acute physiology and chronic health evaluation II; SOFA: Sequential organ failure assessment; PFR: PaO_2_/FiO_2_ ratio; HFNC: High-flow nasal cannula; ICU: Intensive care unit; CKMB: Creatine kinase myocardial band; NL ratio: Neutrophil-to-lymphocyte ratio; ProBNP: Pro-brain natriuretic peptide; CRP: C-reactive protein. * indicates a p-value < 0.05; ** indicates a p-value < 0.05 after Holm's correction for multiple comparison.

Variable	Units	Non-survivors (n = 30)	Survivors (n = 71)	p-value	p-value after Holm's correction
Demographic parameters					
Age (mean [SD])	years	54.70 (12.47)	53.27 (12.88)	0.607	1
Female gender (%)		10 (33.3)	30 (42.3)	0.539	1
APACHE II score on ICU admission (median [IQR])		11.00 [9.25, 12.00]	9.00 [7.00, 11.00]	0.003*	0.131
SOFA score on ICU admission (median [IQR])		4.00 [2.50, 4.00]	2.00 [2.00, 4.00]	0.011*	0.457
Charlson comorbidity index (median [IQR])		2.00 [0.25, 3.00]	1.00 [0.00, 2.50]	0.297	1
Physiological variables and oxygenation status					
Heart rate at baseline (mean [SD])	/min	96.27 (19.25)	92.55 (16.01)	0.318	1
Systolic blood pressure at baseline (mean [SD])	mmHg	129.80 (22.16)	137.20 (22.87)	0.137	1
Diastolic blood pressure at baseline (median [IQR])	mmHg	80.00 [70.00, 83.75]	80.00 [70.00, 89.50]	0.266	1
Respiratory rate at baseline (median [IQR])	/min	37.00 [30.00, 40.00]	30.00 [25.00, 35.50]	0.013*	0.509
PFR at admission (median [IQR])	mmHg	80.50 [70.00, 97.50]	100.00 [80.00, 121.00]	0.01*	0.4
On oxygen by mask or nasal cannula during tocilizumab administration (%)		0 (0.0)	3 (4.2)	0.415	1
On non-invasive ventilation/HFNC during tocilizumab administration (%)		30 (100.0)	67 (94.4)		
On invasive mechanical ventilation during tocilizumab administration (%)		0 (0.0)	1 (1.4)		
PFR on day of tocilizumab administration (median [IQR])	mmHg	77.50 [61.50, 93.39]	100.00 [82.50, 128.58]	<0.001*	0.015**
Time course of illness and therapy					
Day of illness at hospital admission (median [IQR])		6.00 [4.25, 10.00]	6.00 [4.00, 7.00]	0.479	1
Day of illness at ICU admission (median [IQR])		9.00 [6.00, 12.00]	7.00 [6.00, 9.00]	0.056	1
Day of illness at tocilizumab administration (median [IQR])		10.00 [7.00, 13.75]	8.00 [6.00, 9.00]	0.019*	0.689
Number of patients receiving second dose of tocilizumab (%)		22 (73.3)	66 (93.0)	0.018*	0.917
Day of illness at discharge or death (median [IQR])		21.00 [16.25, 31.00]	20.00 [17.50, 24.50]	0.517	1
Day of illness at intubation (median [IQR])		14.00 (n = 2) [11.25, 17.75]	8.50 (n = 29) [7.75, 9.25]	0.061	1
Duration of mechanical ventilation (median [IQR])	days	7.50 [4.00, 11.00]	7.00 [7.00, 7.00]	0.876	1
Length of ICU stay (median [IQR])	days	13.00 [9.25, 21.75]	9.00 [6.50, 12.00]	0.001*	0.041**
Investigations at baseline					
Hemoglobin (median [IQR])	gm/dL	12.00 [11.00, 13.30]	12.00 [11.00, 12.95]	0.858	1
Total leukocyte count (median [IQR])	×1000 /mL	9.25 [7.18, 12.95]	9.50 [7.35, 11.60]	0.603	1
Platelet count (median [IQR])	×1000 /mL	208.00 [166.50, 248.25]	222.00 [145.00, 272.00]	0.677	1
Activated partial thromboplastin time (median [IQR])	seconds	30.00 [27.00, 30.88]	30.50 [27.95, 32.55]	0.199	1
International normalized ratio (median [IQR])		1.10 [1.01, 1.16]	1.10 [1.07, 1.20]	0.357	1
Serum D-dimer (median [IQR])	×1000 ng/mL	1.99 [0.92, 3.23]	0.76 [0.57, 1.48]	0.002*	0.079
Serum fibrinogen (median [IQR])	g/L	7.40 [6.52, 8.10]	7.51 [5.78, 9.00]	0.975	1
Serum albumin (median [IQR])	g/dL	3.30 [3.10, 3.50]	3.31 [3.12, 3.54]	0.449	1
Serum aspartate aminotransferase (median [IQR])	U/L	56.60 [43.50, 81.75]	51.00 [33.40, 69.00]	0.265	1
Serum alanine transaminase (median [IQR])	U/L	49.50 [35.50, 62.70]	47.00 [32.60, 66.10]	0.451	1
Serum alkaline phosphatase in (mean [SD])	U/L	117.26 (50.40)	116.38 (44.89)	0.934	1
Serum urea (median [IQR])	mg/dL	49.50 [32.25, 59.25]	45.00 [30.95, 57.50]	0.38	1
Serum creatinine (median [IQR])	mg/dL	0.72 [0.64, 0.86]	0.76 [0.64, 0.86]	0.885	1
Serum lactate (median [IQR])	mmol/L	1.71 [1.60, 2.17]	2.00 [1.94, 2.50]	0.014*	0.523
Serum lactate dehydrogenase (median [IQR])	×100 U/L	6.68 [5.64, 7.92]	5.07 [4.15, 6.35]	<0.001*	0.008**
CKMB (median [IQR])	×10 U/L	3.09 [2.62, 3.88]	2.27 [1.79, 3.05]	<0.001*	0.003**
NL ratio (median [IQR])	×10	2.47 [1.52, 3.30]	1.00 [0.78, 1.49]	<0.001*	0.001**
ProBNP (median [IQR])	×100 pg/mL	4.20 [3.01, 11.45]	2.59 [1.45, 5.53]	0.002*	0.076
Serum troponin T (median [IQR])	×10 pg/mL	1.21 [0.91, 4.08]	0.78 [0.40, 1.65]	0.006*	0.233
Serum CRP (median [IQR])	×100 mg/dL	1.38 [0.92, 1.88]	1.01 [0.78, 1.51]	0.028*	1
Serum ferritin (median [IQR])	×100 ng/mL	9.32 [6.78, 14.64]	6.39 [3.82, 10.09]	0.002*	0.108
Serum procalcitonin (median [IQR])	ng/mL	0.13 [0.04, 0.25]	0.14 [0.07, 0.33]	0.234	1

Among individual variables, on ROC analysis, NL ratio with a cut-off of 15.8 (AUROC 0.770, specificity 0.79, sensitivity 0.73); CKMB with a cut-off of 25.7 U/L (AUROC 0.751, specificity 0.66, sensitivity 0.87); LDH with a cut-off of 521 U/L (AUROC 0.738, specificity 0.59, sensitivity 0.83), and PFR on day of tocilizumab administration with an individual cut-off of 97.5 mmHg (AUROC 0.727, specificity 0.61, sensitivity 0.80) offered best discrimination of mortality.

The best logistic regression model identified on the basis of genetic algorithm-based screening and manual verification to predict the outcome of death incorporated LDH, NL ratio, ProBNP, and PFR on the day of tocilizumab administration. Stepwise backward elimination of predictors was done as a sensitivity analysis; however, all the selected parameters were retained as removal of any one of the predicting parameters worsened the model performance (Table 2).

**Table 2:** Coefficients and model performance parameters for the selected multivariate logistic regression model (A) and two other candidate models (B and C) with stepwise removal of least significant predictors It can be seen that any further removal of predictors worsens the model performance. ProBNP: Pro-brain natriuretic peptide; PFR: PaO_2_/FiO_2_ ratio; AIC: Akaike information criterion; C index: Area under the receiver operating characteristics curve. * indicates p < 0.1; ** indicates p < 0.05; *** indicates p < 0.01.

Predictors	Model A Mortality ~ LDH100 + NL10 + ProBNP100 + PFR_TOCI	Model B Mortality ~ LDH100 + NL10 + PFR_TOCI	Model C Mortality ~ LDH100 + NL10
	Coefficients		
Serum lactate dehydrogenase/100 (in U/L) LDH100	0.283** (0.055, 0.512)	0.289** (0.063, 0.516)	0.329*** (0.111, 0.546)
Neutrophil-to-leukocyte ratio/100 NL10	0.565** (0.123, 1.006)	0.577** (0.137, 1.017)	0.677*** (0.251, 1.103)
ProBNP/100 (in pg/ml) ProBNP100	0.048 (-0.012, 0.107)		
PFR on day of tocilizumab administration (in mmHg) PFR_TOCI	-0.021** (-0.041, -0.001)	-0.019** (-0.038, -0.001)	
Constant	-2.004 (-4.612, 0.604)	-1.863 (-4.428, 0.703)	-4.122*** (-5.808, -2.437)
Model performance parameters			
R^2^	0.422	0.380	0.328
Chi^2^	35.562*** (df = 4)	31.401*** (df = 3)	26.483*** (df = 2)
AIC	97.32	99.48	102.40
C index	0.855	0.837	0.808

**Figure 1:**
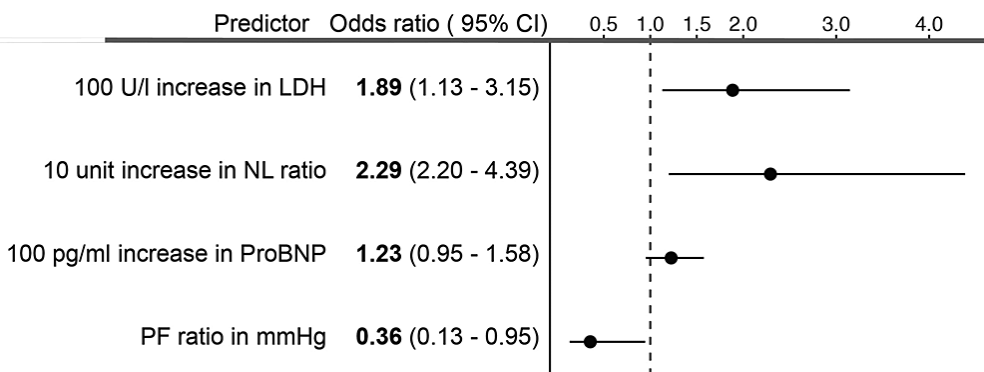
Forest plot showing estimated odds ratios for each of the selected variables along with the 95% confidence intervals LDH: Lactate dehydrogenase; NL ratio: Neutrophil-to-leukocyte ratio; ProBNP: Pro-brain natriuretic peptide; P/F ratio: PaO_2_/FiO_2_ ratio.

**Figure 2:**
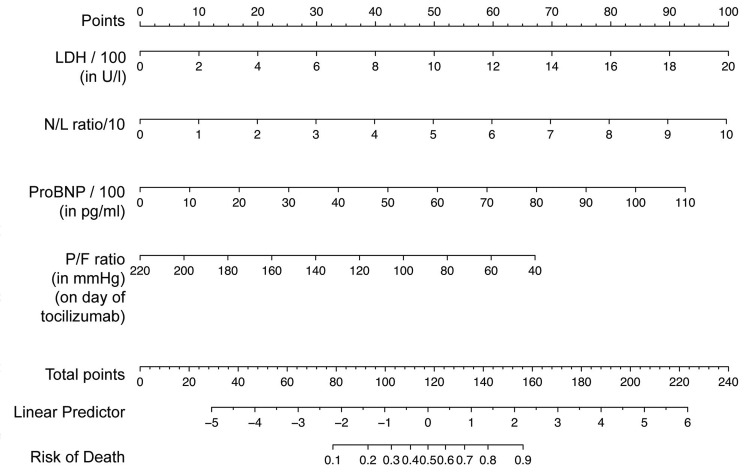
A nomogram for the estimation of probability of mortality following administration of tocilizumab using the selected multivariate logistic regression model LDH: Lactate dehydrogenase; N/L ratio: Neutrophil-to-leukocyte ratio; ProBNP: Pro-brain natriuretic peptide; P/F ratio: PaO_2_/FiO_2_ ratio.

The selected model had an adjusted R^2^ of 0.422 and AUROC (C index) of 0.855 as fitted in our cohort (Table 2, Model A). The estimated odds ratios for mortality for each of the model variables along with confidence intervals are shown in Figure 1. This indicated a 100 U/L increase in LDH or a 10 unit increase in NL ratio; both individually increased the odds of mortality almost two-fold. A nomogram (Figure 2) was created to estimate the probability of mortality using the model parameters.

**Figure 3:**
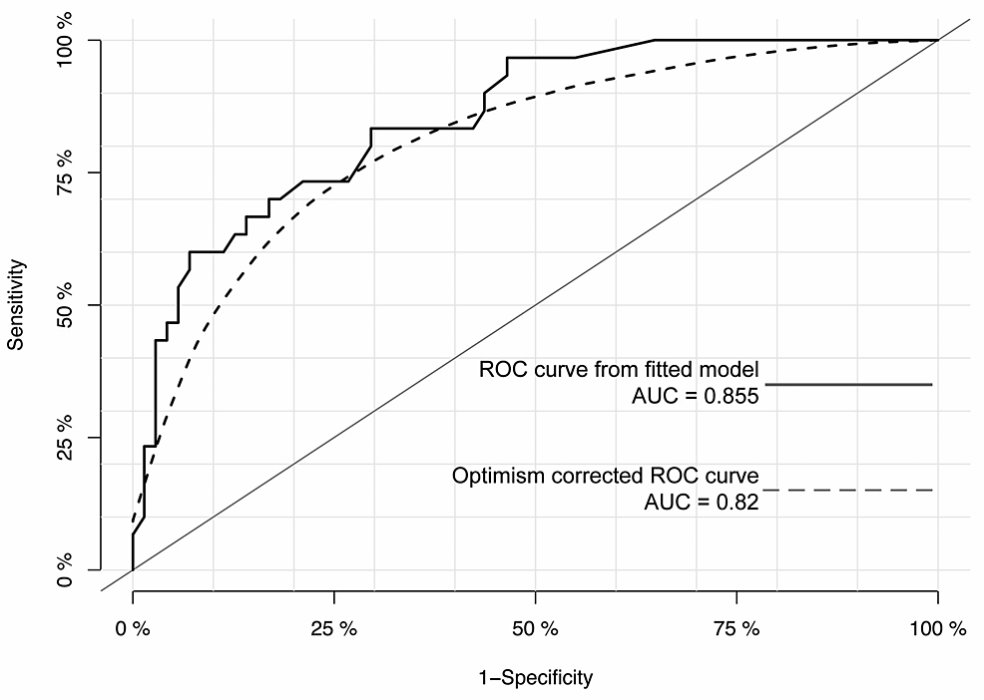
ROC curves based on the model as fitted and after internal validation by 500-fold bootstrapping The minimal difference between the area under the two curves (0.855 and 0.82) indicates insignificant overfitting. ROC: Receiver operating characteristics; AUC: Area under the curve.

Finally, a 500-fold bootstrapping with cross-validation in our cohort was used to validate the selected model. This estimated a corrected area under the curve (AUC) of 0.82, indicating the moderate discriminative performance of the model and insignificant overfitting (Figure 3).

## Discussion

The role of tocilizumab in COVID-19 pneumonia has been controversial. The REMAP-CAP trial, though exhibiting benefits with interleukin-6 inhibitors, had a mortality of 27% among patients receiving tocilizumab or sarilumab [9]. In the large multicenter STOP-COVID trial, tocilizumab was associated with a reduction in mortality compared to the standard of care group, though 125 out of the 433 patients treated with tocilizumab died [12]. The meta-analysis by Malgie et al. also indicated a mortality of 27%, and a number needed to treat of 11 to prevent mortality using tocilizumab in addition to usual care. We have observed a similar rate of mortality in our cohort of patients treated with tocilizumab [10].

This indicates that though there might be a clinical benefit in using tocilizumab in severe cases, there is a cohort of patients where the therapy is likely to fail. Many past works have tried to identify the predictors for failure of tocilizumab therapy. The commonly used biomarkers include levels of interleukin-6, CRP, LDH, D-dimer, etc. [13-17]. Time points of tocilizumab use, SOFA score, etc. were also significantly different in survivors and non-survivors [18]. These mirror the differences observed in our cohort as well.

We have also attempted to prepare a logistic regression model to predict the worse clinical outcomes following the treatment with tocilizumab among critically ill COVID-19 patients. The model includes simple parameters available at baseline: NL ratio, LDH, CKMB, and PFR on the day of administration of tocilizumab. It was shown to predict the chances of treatment failure with acceptable discrimination. A nomogram-based approach to multivariate modeling allows easy clinical prediction and risk stratification. As an example, a patient with a baseline LDH of 1000, NL ratio of 20, ProBNP of 6000, and a PFR of 140 on the day of tocilizumab administration has 50 + 20 + 50 + 30 = 150 points according to the nomogram, indicating a probability of between 0.8 and 0.9 of mortality.

Many predictive models have been created in the context of COVID-19 pneumonia predicting disease severity, progression, or risk of intensive care admission [19-22]. Mussini et al. also tried to identify and model the predictors for tocilizumab failure. Their model reached a cross-validation-corrected AUC of 0.87 for a composite outcome measure of being on mechanical ventilation or mortality on the 28th day following the initiation of treatment, including gender, CRP, baseline platelet count, and PFR on the fourth day following tocilizumab administration [23]. However, the standard of care in their study cohort was hydroxychloroquine, lopinavir, and anticoagulation, which does not reflect current practice. It also includes a predictor measured on the fourth day, delaying prediction. We aimed to study the predictors of tocilizumab among the baseline variables in a clinically more relevant cohort (as indicated by lower admission PFR in our cohort), using steroids and anticoagulation as our standard of care as supported by current evidence [4]. Our model shows comparable discrimination to their model in terms of AUC derived by internal validation through bootstrapping.

Our study was limited in being a single-center study with limited sample size and lacking an external validation cohort. Though internal validation with bootstrapping was done, the results should be reevaluated prospectively in larger cohorts. Indications for tocilizumab administration are still unclear, and the availability of tocilizumab is often sparse in a pandemic situation [24]. An optimization and extension of this model may help in the early identification of patients who have the most survival benefit from tocilizumab and aid in early triage and initiation of rescue therapies.

## Conclusions

High NL ratio, high serum LDH level, high serum CKMB level, and low PaO_2_/FiO_2_ ratio at the time of intervention are important univariate predictors of risk of treatment failure following tocilizumab therapy. A multivariable logistic regression model including serum LDH level, NL ratio, serum ProBNP level, and PaO_2_/FiO_2_ ratio at the time of intervention best predicted the risk of mortality in tocilizumab-treated patients. Further prospective or retrospective evaluation of the predictive performance of this model in larger cohorts may allow a better understanding of the factors influencing response to tocilizumab.

## References

[1] (2022). WHO coronavirus (COVID-19) dashboard. https://covid19.who.int..

[2] Biran N, Ip A, Ahn J (2020). Tocilizumab among patients with COVID-19 in the intensive care unit: a multicentre observational study. Lancet Rheumatol.

[3] Cavalli G, De Luca G, Campochiaro C (2020). Interleukin-1 blockade with high-dose anakinra in patients with COVID-19, acute respiratory distress syndrome, and hyperinflammation: a retrospective cohort study. Lancet Rheumatol.

[4] Horby P, Lim WS, Emberson JR (2021). Dexamethasone in hospitalized patients with COVID-19. N Engl J Med.

[5] Rosas IO, Bräu N, Waters M (2021). Tocilizumab in hospitalized patients with severe COVID-19 pneumonia. N Engl J Med.

[6] Stone JH, Frigault MJ, Serling-Boyd NJ (2020). Efficacy of tocilizumab in patients hospitalized with COVID-19. N Engl J Med.

[7] Salvarani C, Dolci G, Massari M (2021). Effect of tocilizumab vs standard care on clinical worsening in patients hospitalized with COVID-19 pneumonia: a randomized clinical trial. JAMA Intern Med.

[8] Salama C, Han J, Yau L (2021). Tocilizumab in patients hospitalized with COVID-19 pneumonia. N Engl J Med.

[9] Gordon AC, Mouncey PR, Al-Beidh F (2021). Interleukin-6 receptor antagonists in critically ill patients with COVID-19. N Engl J Med.

[10] Malgie J, Schoones JW, Pijls BG (2021). Decreased mortality in coronavirus disease 2019 patients treated with tocilizumab: a rapid systematic review and meta-analysis of observational studies. Clin Infect Dis.

[11] Huang H, Cai S, Li Y (2020). Prognostic factors for COVID-19 pneumonia progression to severe symptoms based on earlier clinical features: a retrospective analysis. Front Med (Lausanne).

[12] Gupta S, Wang W, Hayek SS (2021). Association between early treatment with tocilizumab and mortality among critically ill patients with COVID-19. JAMA Intern Med.

[13] Galván-Román JM, Rodríguez-García SC, Roy-Vallejo E (2021). IL-6 serum levels predict severity and response to tocilizumab in COVID-19: an observational study. J Allergy Clin Immunol.

[14] Khurshid S, Rehman N, Ahmed S (2021). Early fall in C-reactive protein (CRP) level predicts response to tocilizumab in rapidly progressing COVID-19: experience in a single-arm Pakistani center. Cureus.

[15] Conrozier T, Lohse A, Balblanc JC (2020). Biomarker variation in patients successfully treated with tocilizumab for severe coronavirus disease 2019 (COVID-19): results of a multidisciplinary collaboration. Clin Exp Rheumatol.

[16] De Ardanaz SL, Andreu-Ubero JM, Navidad-Fuentes M (2021). Tocilizumab in COVID-19: factors associated with mortality before and after treatment. Front Pharmacol.

[17] Desai HD, Sharma K, Parikh A (2021). Predictors of mortality amongst tocilizumab administered COVID-19 Asian Indians: a predictive study from a tertiary care centre. Cureus.

[18] Morrison AR, Johnson JM, Griebe KM (2020). Clinical characteristics and predictors of survival in adults with coronavirus disease 2019 receiving tocilizumab. J Autoimmun.

[19] Guan X, Zhang B, Fu M (2021). Clinical and inflammatory features based machine learning model for fatal risk prediction of hospitalized COVID-19 patients: results from a retrospective cohort study. Ann Med.

[20] Xue G, Gan X, Wu Z (2020). Novel serological biomarkers for inflammation in predicting disease severity in patients with COVID-19. Int Immunopharmacol.

[21] Fan T, Hao B, Yang S (2020). Nomogram for predicting COVID-19 disease progression based on single-center data: observational study and model development. JMIR Med Inform.

[22] Guner R, Kayaaslan B, Hasanoglu I (2021). Development and validation of nomogram to predict severe illness requiring intensive care follow up in hospitalized COVID-19 cases. BMC Infect Dis.

[23] Mussini C, Cozzi-Lepri A, Menozzi M (2021). Development and validation of a prediction model for tocilizumab failure in hospitalized patients with SARS-CoV-2 infection. PLoS One.

[24] Raiteri A, Piscaglia F, Granito A, Tovoli F (2021). Tocilizumab: from rheumatic diseases to COVID-19. Curr Pharm Des.

